# Quantifying Head Impacts in Elite Muay Thai: A Case Study Using Instrumented Mouthguards

**DOI:** 10.3390/sports14030111

**Published:** 2026-03-11

**Authors:** Luke Del Vecchio, Mike Climstein, Daniel A. Brown

**Affiliations:** 1 Faculty of Health, Southern Cross University, Gold Coast, QLD 4225, Australia; michael.climstein@scu.edu.au (M.C.); daniel.brown@scu.edu.au (D.A.B.); 2 Physical Activity, Lifestyle, Ageing and Wellbeing Faculty Research Group, University of Sydney, Sydney, NSW 2141, Australia

**Keywords:** combat sports, head impacts, sensor-equipped mouthguards, athlete monitoring

## Abstract

Instrumented mouthguards (iMGs) enable in vivo monitoring of head-impact exposure by reporting event-level peak linear acceleration (PLA) and peak angular acceleration (PAA) in contact sports. This case study describes head impacts in a world-class Muay Thai fighter during routine sparring sessions over a two-week period leading into a competitive bout. Seven sparring sessions were monitored using an iMG (PROTeQT, HitIQ), and only manufacturer (in-mouth)-flagged events above the device’s 8 g trigger threshold were analyzed. Event-level data were exported from the manufacturer portal; raw time-series signals and proprietary signal-processing parameters were not accessible, and no independent video verification was performed. Across the camp, 590 impacts were recorded. Mean PLA values were modest across sessions (7.6 to 19.5 g), with one event exceeding 106 g (max PLA 162.2 g). In contrast, PAA exhibited greater variability, with multiple device-flagged events exceeding 7900 rad/s^2^, particularly in Sessions 4, 6, and 7, where maximum PAA values reached 19,862 to 26,850 rad/s^2^. Overall, these data indicate that sparring was predominantly low in translational loading, while occasionally producing high recorded rotational peaks. Because outputs are device- and processing-pipeline-specific and were not independently verified, threshold-based severity banding and extreme peaks should be interpreted cautiously. This case demonstrates the potential utility of iMG monitoring to characterize session-to-session variability in sparring exposure and to inform practical sparring load management strategies aimed at reducing cumulative head-impact burden.

## 1. Introduction

Sport-related concussion and broader head-impact exposure are now understood primarily through a biomechanical lens. The most recent international consensus statement describes sport-related concussion as a traumatic brain injury induced by biomechanical forces, with heterogeneity in clinical presentation and recovery. It also underscores the ongoing need for improved exposure quantification, risk reduction, and individualised management [1]. Similarly, the American Medical Society for Sports Medicine position statement emphasises that concussion risk and outcomes reflect complex interactions among biomechanics, athlete factors, and context, and that improved prevention requires multi-layer strategies rather than reliance on any single metric [2].

Concerns extend beyond diagnosed concussions to RHI exposure over time. Neuropathology case series and reviews describe chronic traumatic encephalopathy (CTE) as a tauopathy associated with repetitive mild traumatic brain injury and RHI exposure, with characteristic patterns of hyperphosphorylated tau deposition and potential co-pathology [3,4,5]. Epidemiological studies in contact-sport populations (e.g., former professional soccer players) report higher rates of neurodegenerative disease outcomes compared with population controls, although causality, sport specificity, and confounding remain active scientific issues [6,7]. These studies collectively motivate monitoring approaches that consider not only “concussion counts” but also cumulative head-impact exposure and the mechanical nature of those impacts.

A key reason that the “mechanical nature” of impacts matters is that brain tissue injury mechanisms differ for translational versus rotational head motion. Brain tissue is nearly incompressible, so translation tends to generate pressure gradients that may be more relevant to focal injury modes, whereas rotation generates shear deformation that is strongly implicated in diffuse tissue strain and axonal injury mechanisms [8,9]. Consistent with this, conceptual work and modern computational and finite element analyses indicate that rotational kinematics are central determinants of diffuse brain deformation in many impact scenarios [8,9]. Practically, two impacts with similar peak linear acceleration can differ meaningfully in injury-relevant brain strain when peak angular acceleration, rotational velocity, or impact duration differ.

Within finite element (FE) biomechanics, head kinematics can be propagated through validated head models to estimate brain tissue responses such as maximum principal strain (MPS) and strain rate, often summarised using high-percentile strain values (e.g., 95th percentile MPS) to reduce sensitivity to extreme single-element peaks [10,11,12]. FE-derived strain has been used both to interpret concussion mechanisms and to develop rapid surrogate metrics that approximate brain strain from kinematics [10,11,12]. For example, the Diffuse Axonal Multi-Axis General Evaluation (DAMAGE) metric represents brain deformation response to rotational head motion as a second-order system and aims to estimate maximum brain strain from multi-axis angular acceleration time histories [13]. These approaches are important because “impact severity” is not well captured by any single scalar measure (e.g., peak linear acceleration alone), and because concussion risk curves show substantial overlap between concussive and non-concussive impacts.

Injury-risk curve work in instrumented American football cohorts illustrates this probabilistic nature. Rowson and colleagues derived a combined probability of concussion metric using both peak linear and peak rotational accelerations, based on large numbers of instrumented impacts and clinically identified concussions, and showed that combining kinematic domains improved predictive performance in some datasets [14]. Separately, combat-sport and reconstruction/modelling work has proposed illustrative probabilistic thresholds (e.g., 25%, 50%, 80% probability of mild traumatic brain injury) at paired PLA and PAA values, commonly described as 66 g/4600 rad/s^2^, 82 g/5900 rad/s^2^, and 106 g/7900 rad/s^2^, while acknowledging uncertainty and transferability between sports and methods [15]. These thresholds are best interpreted as risk–framework anchors, not diagnostic cut-points, particularly when used outside the sport/model context in which they were developed. Moreover, when severity bands are applied to field data, interpretation depends critically on the integrity of the measured signal and the processing pipeline used to derive the reported peaks and event classifications.

Modern iMG systems can support this biomechanical interpretation by capturing in vivo head kinematics during sport participation. The iMG approach has a strong methodological rationale: sensors fixed to the upper dentition can provide improved coupling to the skull compared with soft-tissue locations, reducing motion artefact and enabling measurement of both translational and rotational kinematics [16,17,18]. Laboratory validation studies report high agreement between iMG outputs and headform reference sensors across broad ranges of impact magnitudes. For example, Stitt et al. reported a strong correlation and agreement between a HitIQ instrumented mouthguard and headform reference sensors across multiple impact locations and intensities [18]. Jones et al. evaluated an iMG across a wide range of sport-relevant accelerations and demonstrated close agreement in FE-predicted brain strain between iMG-derived kinematics and reference measurements [11].

However, iMG measurement is not “plug-and-play.” Field performance depends on device algorithms, triggering thresholds, post-processing and labelling rules, athlete compliance (in-mouth time), and the presence or absence of synchronised video verification [19]. Importantly, many commercial iMG systems implement proprietary signal-processing, filtering, event-detection logic, and artefact-rejection steps that are not fully disclosed. As a result, reported head-impact counts, peak values, and derived “severity” classifications should be interpreted as device- and pipeline-specific outputs rather than as universally comparable measures of true head kinematics. This limitation is particularly relevant when interpreting the tail of the distribution, because without independent verification, it cannot be assumed that a flagged high-magnitude event represents a true high-magnitude head impact rather than an artefact, coupling irregularity, or processing-related peak.

World Rugby’s iMG specifications formalise this reality by requiring performance criteria in laboratory testing and in on-field testing (e.g., positive predictive value ≥90% and sensitivity ≥80% for detecting direct head impacts), with continuous video as part of field evaluation [20]. Video-verified field studies illustrate both the potential and the need for such validation: for example, a rugby union pilot study reported sensitivity 93.6% and positive predictive value (PPV) 92.4% [21]. Conceptual and methodological reviews also emphasise that iMG “sensor acceleration events” are not identical to true head acceleration events and can be influenced by thresholding and artefact, motivating careful interpretation and transparent processing descriptions [22].

In combat sports, the need for objective monitoring is especially salient because training can involve frequent head contacts and because rotational kinematics may be prominent in unhelmeted striking [23]. iMG-based studies in boxing and mixed martial arts (MMA) show that sparring exposures are typically skewed toward low magnitudes but can involve substantial numbers of impacts per session [15]. A cohort study characterising boxing and MMA impacts reported comparable impacts per athlete per session across sports, with differences in angular acceleration distributions and higher median peak angular acceleration (PAA) in MMA than boxing [15]. MMA studies that included concussive events report substantially higher concussive event kinematics (e.g., average concussive PLA 86.7 g and PAA 7561 rad/s^2^), and finite element simulation work in MMA suggests brain strain metrics (e.g., corpus callosum strain) may discriminate concussive from non-injurious events in that context [22]. Importantly, sport-specific and context-specific differences (rules, glove size, stance, clinch, fatigue, defensive anticipation, and whether impacts are “seen” versus “unseen”) can change both the magnitude and direction of head loading. In addition, instrumented mouthguard outputs can be affected by mandible dynamics and sensor decoupling, particularly under open-mouth or altered coupling conditions [17,24,25]. These considerations further reinforce that large reported peaks in field datasets require careful scrutiny and, where possible, independent verification before being interpreted as unequivocal high-severity head impacts.

Despite Muay Thai’s international popularity and distinctive striking repertoire (punches, kicks, knees, elbows, clinch), peer-reviewed head-impact exposure characterisation remains comparatively limited. A recent Muay Thai case study integrating iMG monitoring with blood biomarkers illustrates feasibility and suggests potential dissociations between different kinematic features and biomarker responses, but broader training-camp exposure profiles in elite-level athletes remain under-described [26]. This gap matters because training–sparring exposures are modifiable through coaching decisions (frequency, intensity, partner matching, technical emphasis) and represent a primary target for preventive strategies that do not require rule changes at the competition level.

In practical terms, the most common head-impact exposures in Muay Thai sparring typically arise from straight punches, hooks, and defensive exchanges during combination drills, whereas higher-magnitude rotational loading may occur during unanticipated hooks, overhand strikes, spinning techniques, or head displacement during clinch disengagement. Kicks and knees that contact the head can also generate substantial angular acceleration due to longer moment arms and greater segment mass. These sport-specific mechanics help contextualise why both linear and rotational kinematics are relevant when interpreting iMG outputs in striking disciplines.

Accordingly, the aim of this case study was to describe and interpret sparring-related head impacts in a world-class Muay Thai fighter during a two-week fight camp using an iMG system. This study was descriptive in nature and did not aim to validate the device, assess agreement with a reference standard, or evaluate diagnostic performance. The analysis focused on both linear and angular kinematics, session-to-session variability, and the implications of rotationally dominated events for training design and risk reduction. In doing so, impact counts and severity bands are treated as device- and processing-pipeline-specific indicators of head-impact exposure, and high-severity classifications are interpreted cautiously in the absence of independent verification.

## 2. Case Description

### 2.1. Participant

The participant was a male Australian Muay Thai fighter competing in the 63.5–65 kg weight class. At the time of monitoring, his most recent recorded fight weight was 65 kg (143 lb). He held a professional record of 8–3–0, with two wins by KO/TKO.

The athlete had competed in recognised promotions, including Rajadamnern World Series, LWC Super Champ, Fairtex Fight, Rebellion Muay Thai, X Fighting Championship, and Muay Thai League. He reported approximately 10 years of competitive experience and no diagnosed concussion within the previous 12 months.

#### Context

Monitoring occurred during preparation for a competitive bout in 2025 and included seven sparring sessions across two consecutive weeks in a professional Muay Thai gym setting.

The athlete trained six times per week, typically comprising morning technical, tactical, or conditioning sessions, and evening sessions focused on sparring or Thai pad work. The monitored sessions represented structured sparring typical of elite Muay Thai preparation, including punch, kick, knee, elbow, and clinch exchanges.

The athlete wore a custom-fitted instrumented mouthguard during sparring. Written informed consent was obtained prior to monitoring, and de-identified data were used for research purposes.

## 3. Materials and Methods

The athlete wore an instrumented mouthguard (iMG; model PROTeQT, HitIQ, Victoria, Australia) during seven sparring sessions conducted over a two-week training period (8 to 22 August 2025). The device contained tri-axial accelerometers and gyroscopes sampling at 1000 Hz, with an 8 g trigger threshold. Only manufacturer-flagged in-mouth events were retained for analysis. The reliability and validity of the HitIQ iMG system have been established in independent laboratory validation studies.

Impact data were accessed via the manufacturer’s secure cloud-based data portal, which serves as a repository and visualisation interface and enables export of event-level outputs. The portal provides session summaries and downloadable event-level variables, including timestamps, peak linear acceleration (PLA), peak angular acceleration (PAA), and manufacturer quality-assurance labels (e.g., in-mouth flagging). The portal was used for data access and export only. No investigator-defined signal processing, filtering choices, coordinate transformations, centre-of-gravity estimation, or other analytical transformations were performed within the platform, and the study analyses were undertaken using exported event-level data.

Seven sparring sessions were monitored, each following standard fight-camp programming consisting of four to six three-minute rounds. No formal sparring-intensity scale was collected. This reflects current practice in Muay Thai, where sparring intensity is typically managed subjectively by the coach and athlete rather than quantified using validated intensity tools or formal guidelines. Accordingly, session intensity was described qualitatively by the athlete as “moderate” to “heavy.”

**Scheme 1:**
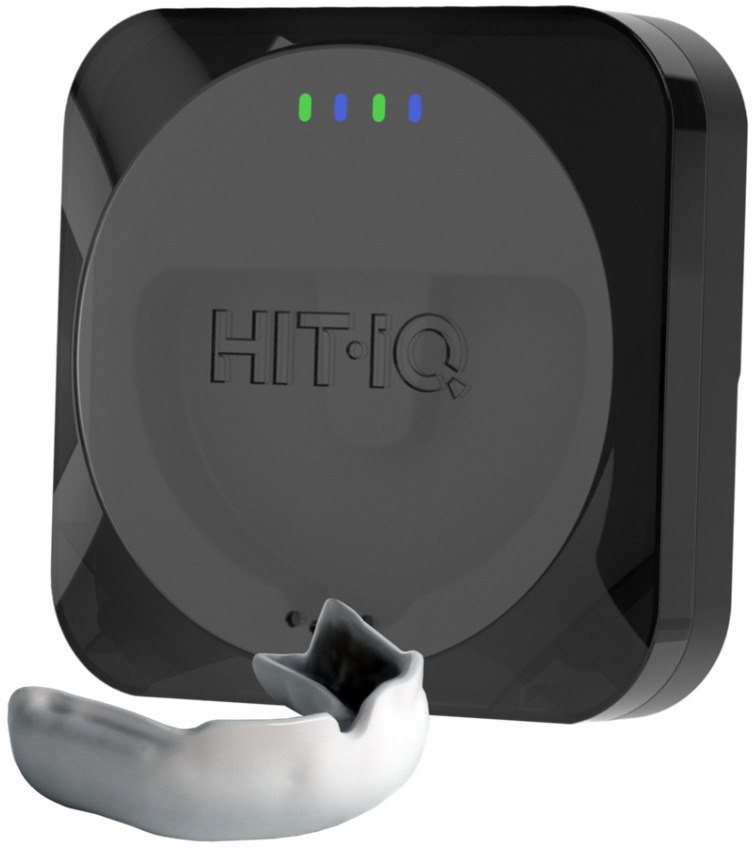
Instrumented mouthguard (HitIQ, Australia) used for head-impact monitoring in this study, shown with its charging case. The device houses embedded accelerometers and gyroscopes and uploads event-level outputs to a secure cloud-based data portal following synchronisation.

In addition, a representative image of the instrumented mouthguard and charging case is provided (Scheme 1) to support transparency regarding the hardware used for head-impact monitoring in this case study.

Scheme 1 An instrumented mouthguard and charging case used for head-impact monitoring in this study.

### 3.1. Signal Processing and Data Cleaning

Impact data were exported from the manufacturer platform at the event level. For each detected event, the exported variables included peak linear acceleration (PLA, g), peak angular acceleration (PAA, rad/s^2^), event timestamp, and session identifier. Device-generated quality labels (including in-mouth classification and automated QA or event flags) were also exported. The 8 g threshold refers to the device’s event trigger. An event is logged when resultant linear acceleration exceeds 8 g, which initiates device recording and processing for that event. The manufacturer then applies automated quality assurance to classify each detected event, including an “in-mouth” label indicating the mouthguard was worn and coupled to the dentition at the time of the event. In this study, “flagged in-mouth events” refers to events that met the 8 g trigger and were labelled by the manufacturer as in-mouth. Events not labelled in-mouth, or otherwise flagged by the platform as poor quality or non-wearable, were excluded. Raw accelerometer and gyroscope time-series data were not accessible to the investigators.

Signal-processing parameters, including filter type and cutoffs, phase characteristics, coordinate frame transformations, head centre-of-gravity estimation, and impact windowing, are proprietary and were not available for reporting. Because peak linear and especially peak angular acceleration estimates are sensitive to filtering and windowing choices, the exported PLA and PAA values should be interpreted as device-specific outputs. Accordingly, analyses in this case study were performed using exported event-level peak metrics only. Severity banding and comparisons with external thresholds are therefore treated as device-specific and not assumed to be directly comparable across studies or sensor systems unless identical processing pipelines are confirmed.

Data cleaning was limited to the manufacturer’s in-mouth classification and exported QA or event labels. No additional investigator-defined filtering or outlier removal was applied. No independent video verification was performed in this case study. Given evidence that video verification can improve event classification and refine positive predictive value, the absence of video verification is treated as a limitation and a priority recommendation for future work [20,21].

### 3.2. Ethics Statement

This project was submitted to the Southern Cross University Low Risk Committee (LRC) for review under the title “Retrospective clinical case study: Instrumented-Mouthguard Surveillance During a Muay Thai World-Title Fight-Camp.” Following review, the Chair determined that the project did not meet the institutional definition of research requiring Human Research Ethics Committee (HREC) approval because it involved a single retrospective clinical case, was not designed to produce generalisable findings, and did not involve experimental manipulation or hypothesis testing.

In accordance with this determination, formal HREC approval was not required. The participant was informed of the intended use of the data for publication, provided consent for de-identified reporting, and all data were stored securely in accordance with institutional data protection policies. A formal record of this determination is retained by the university.

## 4. Results

Across seven sparring sessions, a total of 590 head impacts were recorded by the instrumented mouthguard (iMG). Session-level mean peak linear acceleration (PLA) ranged from 7.6 to 19.5 g, with a maximum PLA of 162.2 g recorded in Session 4. Session-level mean peak angular acceleration (PAA) ranged from 530 to 2784 rad/s^2^, with a maximum PAA of 26,850 rad/s^2^ recorded in Session 6.

Impact severity was classified separately for PLA and PAA using the predefined thresholds, and results are reported using the corresponding units for each domain. For PLA, most events were mild (PLA < 66 g), with one event in the moderate range (66–106 g) and one event in the device-flagged severe range (>106 g). For PAA, most events were mild (PAA < 4600 rad/s^2^), with multiple events in the device-flagged severe range (>7900 rad/s^2^), particularly in Sessions 4, 6, and 7. Table 1 summarizes the session-by-session outcomes for PLA- and PAA-based classifications.

### 4.1. Participant: Distribution of Linear Acceleration (G) Across Sessions

**Figure 1:**
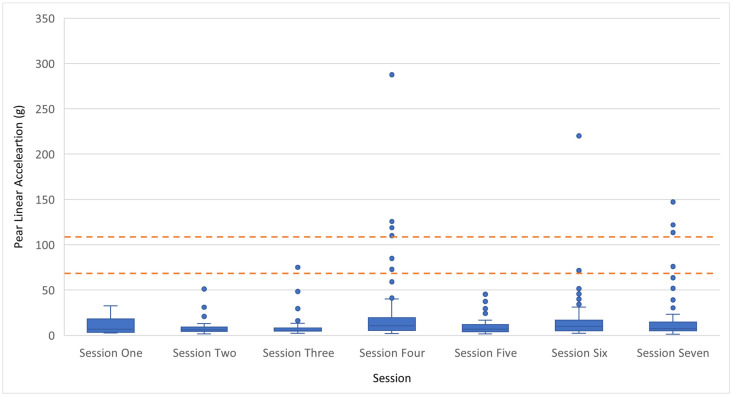
Distribution of peak linear accelerations (PLA, g) across seven sparring sessions. Boxes show the interquartile range (25th–75th percentile) with the median indicated by the horizontal line. Whiskers extend to 1.5× the interquartile range; points represent outliers and maxima, with the maximum corresponding to the highest point per session. Dashed horizontal lines indicate PLA thresholds at 66 g and 106 g.

Most sparring impacts were below commonly cited concussion thresholds for linear acceleration (~95 g). Across all sessions, 98.7% of impacts were <95 g, with Sessions 1–3 and 5 at 100%, Session 6 at 99.2%, Session 4 at 96.7%, and Session 7 at 96.3%. Sessions 2–5 (inclusive) showed low linear accelerations, with 95th percentile values under 40 g and no impacts exceeding 66 g. Session 4 recorded the highest mean PLA (19.5 g) and maximum PLA (162.2 g). This maximum appears to represent an outlier; in Session 4, only one event exceeded the moderate range (66 to 106 g), while the remaining impacts were mild (PLA < 66 g). The distribution of PLA across sessions is shown in Figure 1.

### 4.2. Distribution of Peak Angular Acceleration (rad/s^2^) Across Sessions

**Figure 2:**
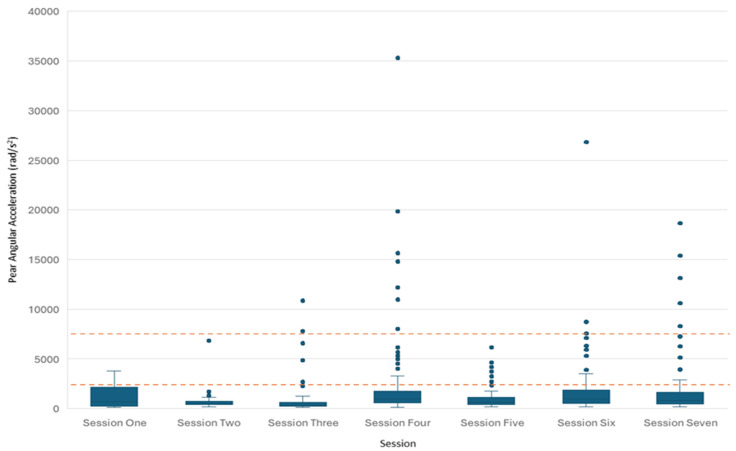
Distribution of peak angular accelerations (PAA, rad/s^2^) across seven sparring sessions. Boxes show the interquartile range (25th–75th percentile) with the median indicated by the horizontal line. Whiskers extend to 1.5× the interquartile range; points represent outliers and maxima, with the maximum corresponding to the highest point per session. Dashed horizontal lines indicate the predefined PAA category boundaries used for severity classification. Sessions 4, 6, and 7 contain multiple events above the upper category boundary.

Across the monitored sessions, most impacts were classified as mild for angular acceleration (PAA < 4600 rad/s^2^), with event-level values spanning from 280 rad/s^2^ up to 9870 rad/s^2^. Sessions 3, 4, 6, and 7 displayed pronounced upper tails, each containing multiple device-flagged severe-range events (PAA > 7900 rad/s^2^). Notably, Session 4 recorded multiple device-flagged severe-range angular-acceleration events despite only one moderate linear-acceleration event in the same session (Figure 2).

### 4.3. Relationship Between Linear and Angular Acceleration

**Figure 3:**
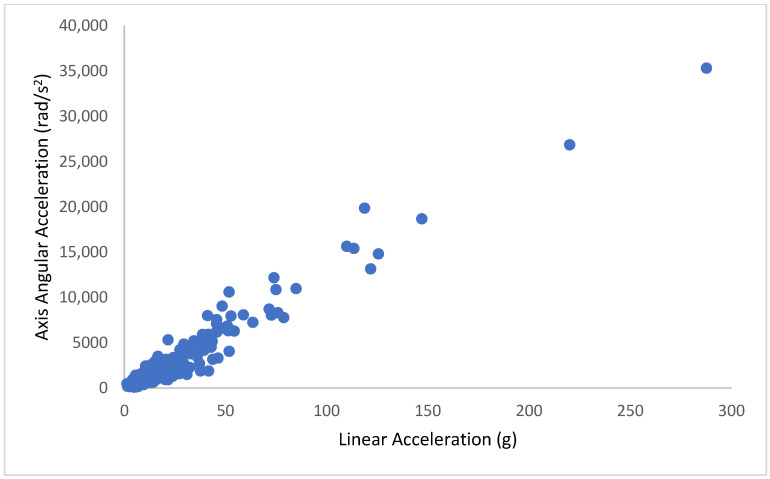
Relationship between peak linear acceleration (g) and peak angular acceleration (rad/s^2^) across all sparring sessions. Higher linear accelerations generally coincided with higher angular accelerations; however, several events show high angular acceleration despite only moderate linear acceleration (upper-left region).

When event-level data from all sessions were pooled, PLA and PAA demonstrated a strong positive association (Pearson r = 0.97; Figure 3). However, several impacts exhibited high angular acceleration (for example, >10,000 rad/s^2^) while the corresponding PLA remained moderate (for example, <40 g). These rotationally dominated events indicate that substantial angular loading can occur even when linear acceleration remains within the mild range.

## 5. Discussion

This case study provides an objective kinematic description of sparring-related head impacts across a two-week fight camp in a world-class Muay Thai fighter using an iMG system. The principal findings are that (i) session mean PLA values were generally low-to-moderate (7.6–19.5 g), comparable to magnitudes reported in combat-sport sparring cohorts; (ii) the session maxima and long-tailed distributions indicate extreme outliers in the recorded signal, particularly for angular acceleration (PAA) with a maximum recorded PAA of 26,850.2 rad/s^2^; and (iii) there was marked session-to-session variability in impacts volume and density, with impacts/min (assumption-based) ranging from <1/min in the lowest-exposure session to >10/min in the highest-exposure session. Importantly, because no independent video verification was available, these findings should be interpreted as device- and processing-pipeline-specific outputs rather than confirmed head-impact magnitudes, and the extreme peaks should be treated as potentially artefactual until corroborated.

The observed mean PLA range in this case (7.6 to 19.5 g) is consistent with the general pattern reported in sparring-focused combat-sport datasets, namely, that distributions are right-skewed with most verified impacts at relatively low magnitudes and fewer high-magnitude events. In a mixed boxing and MMA dataset, sparring impacts had a median PLA of 17.9 g and a median PAA of 1704 rad/s^2^, while competition impacts had higher medians (PLA 21.9 g; PAA 2248 rad/s^2^) [15]. Session mean PAA values in the present case (530 to 2784 rad/s^2^) overlap these ranges, and the higher-PAA sessions (e.g., Session 4 mean PAA 2784 rad/s^2^) are comparable to the magnitude of PAA observed in competition medians in that cohort study [14]. However, without video review, we cannot determine whether the highest recorded PAA peaks represent true head kinematics, glove or guard contacts, jaw-related artefact, or other non-impact events.

However, the frequency of impacts recorded per session in this case (10–129; total 590 across seven sessions) appears higher than the impacts per athlete per session values reported in the boxing/MMA cohort study (boxing 14.0 ± 12.0; MMA 15.3 ± 17.7), and also higher than the male averages reported separately for boxing and MMA sparring sessions (male boxing 18.4 ± 13.1 impacts/session; male MMA 19.0 ± 20.5 impacts/session) [15]. There are several plausible explanations that do not require assuming true differences in exposure: (i) differences in what constitutes a true-positive event (video-verified versus sensor-only), (ii) differences in triggering thresholds and device classification pipelines, (iii) different sparring rulesets, athlete styles, and partner behaviours, and (iv) the potential for false positives when video verification is not used. The present case used manufacturer in-mouth/QA classifications but did not include independent video verification, which World Rugby and methodological guidance identify as important for quantifying PPV and sensitivity in practice [20,21]. Accordingly, the absolute event counts and the frequency of high-category events should be viewed as provisional, pending independent confirmation.

A key contribution of this case is the documentation of very high recorded peak angular acceleration (PAA) outliers alongside generally modest mean peak linear acceleration (PLA) values. This matters because biomechanical evidence indicates that rotational kinematics are closely linked to diffuse brain deformation mechanisms, with brain tissue deforming predominantly in shear due to its material properties and constraints within the skull [8,9]. Consistent with this, conceptual work and computational and finite element analyses reinforce that rotational kinematics are central determinants of predicted brain strain across many impact scenarios [8,9]. At the same time, the present dataset cannot establish that the most extreme PAA values reflect true high-magnitude head impacts, because the absence of video verification limits our ability to rule out iMG artefacts and coupling-related error.

Within finite element (FE) modelling studies, head kinematic time histories are used to estimate tissue-level responses such as maximum principal strain (MPS) and strain rate, and these are commonly summarized using high-percentile strain metrics (e.g., 95th percentile MPS, MPS95) to reduce sensitivity to extreme single-element peaks [10,12,13]. Practically, impacts with similar peak linear acceleration (PLA) can translate into different strain outcomes when rotational kinematics and impact duration differ [12,13]. Consistent with this, modelling work and strain-prediction frameworks emphasize that rotational kinematics, particularly when represented as full time histories rather than single peaks, can be dominant determinants of predicted brain deformation, reinforcing the rationale for monitoring both linear and rotational domains [13]. Because the present study reports peak values rather than raw time-series kinematics, and because processing details are device-specific, any downstream strain-related interpretation should be considered illustrative rather than definitive.

In combat sports specifically, instrumented MMA work that included concussive events reported an average concussive PLA of 86.7 g and an average concussive PAA of 7561 rad/s^2^, and FE simulation work in MMA suggested that corpus callosum strain and shear stress may discriminate concussive from uninjured impacts in that cohort [22,27]. While it would be inappropriate to directly map the present case’s PAA maxima to concussion likelihood because (i) concussion diagnosis data are not presented, (ii) the present dataset reflects training sparring rather than competition, (iii) device hardware and processing pipelines may differ, and (iv) concussion risk curves overlap substantially, this comparison highlights that the maximum PAA recorded here (26,850 rad/s^2^) exceeds the mean concussive PAA reported in that MMA series [22]. This should not be interpreted as evidence that a concussive-level impact occurred in this athlete. Rather, it indicates that the device recorded occasional extreme peaks that warrant cautious interpretation and, ideally, independent verification before being treated as true high-magnitude head impacts. This reinforces the importance of contextual verification (e.g., video) and careful artefact consideration when unusually high PAA spikes occur [20].

A common temptation in applied settings is to treat single-number thresholds as diagnostic boundaries. The broader literature argues against this. Concussion risk curves based on in vivo helmeted sport impacts show substantial overlap between concussive and non-concussive impacts and demonstrate improved predictive utility when linear and rotational accelerations are considered together [14,23]. Combat-sport literature likewise uses probabilistic anchors as a framework for severity staging rather than definitive diagnostic thresholds, and it emphasises that proposed injury thresholds should not be used in the absence of clinical assessment. In this study, threshold bands should be interpreted primarily as a descriptive framework for summarizing device outputs, not as confirmation that an impact was truly of high severity.

In the present case, most impacts appeared to fall below the higher-probability anchors by inference from the low session means and distribution descriptions, while isolated events exceeded these anchors. The appropriate interpretation is therefore not “concussion occurred” but rather: the athlete experienced occasional device-flagged high-magnitude kinematic events during routine sparring, which may be relevant to both acute risk management and chronic exposure optimisation, and which are potentially modifiable through training design. Given the lack of video verification, the more defensible inference is that exposure varied substantially across sessions, and that some sessions contained a small number of extreme recorded peaks that should be treated as uncertain in magnitude until corroborated.

Even if most impacts are individually low-to-moderate, the cumulative burden of repetitive head impacts (RHI) may be relevant for long-term brain health outcomes. Neuropathology reviews and clinicopathological series describe CTE as associated with repetitive head impacts and emphasise the importance of exposure characterisation rather than reliance on concussion diagnosis alone [3,4,5]. Epidemiological studies and systematic reviews in contact sports report elevated neurodegenerative outcomes in some cohorts, motivating preventive approaches that reduce cumulative exposure where possible [7,28]. However, the present case study cannot quantify true RHI dose with high certainty because it lacks independent confirmation of impact validity and magnitude at the event level.

From a practical sports-science standpoint, this motivates treating head-impact exposure as an “external brain load” analogous to how GPS-derived running loads or collision counts are treated as external load in team sports. World Rugby’s deployment of iMGs explicitly frames iMG outputs as a tool to quantify head acceleration event frequency and magnitude to inform management of chronic load [20]. Recent boxing sparring research also suggests that session-level exposure summaries (including cumulative sums and time-weighted metrics) may align better with symptom changes than single-peak measures in some athletes, reinforcing the potential value of richer exposure modelling rather than simplistic thresholds [17]. In this context, session-level trends and relative comparisons (between sessions within the same athlete using the same device and settings) are likely more defensible than strong claims based on isolated extreme peaks.

In this case, the cumulative load proxies (Table 1) show that sessions with the highest impact counts and higher mean PAA (Sessions 4, 6, and 7) contributed disproportionately to the estimated cumulative angular exposure. For example, Session 4’s cumulative PAA proxy (342,432 rad/s^2^·impact) exceeded Session 2’s (52,960 rad/s^2^·impact) by 6.5-fold, driven by both higher mean PAA and higher impact volume. While these proxies do not replace event-level cumulative metrics (and are sensitive to missing information), they illustrate why session-by-session monitoring can be actionable: coaches can identify “high brain-load days” and adjust subsequent training to manage accumulation across weeks. These interpretations remain conditional on the assumption that the recorded events reflect true impacts and that artefactual detections are not dominating specific sessions.

A critical methodological point, particularly for striking sports, is the influence of jaw mechanics and mouthguard coupling on measured kinematics [24,25]. Laboratory evidence shows that different mandible constraints can alter mouthguard measurement accuracy, and recent open-access work demonstrates that open-mouth or decoupling conditions can increase errors in mouthguard kinematic measurements (including properly fit boil-and-bite mouthguards) [24,25]. The present case narrative noted that an unusually high PLA spike may have been influenced by an open-mouth posture combined with clenching during an uppercut impact. This interpretation is consistent with the broader literature: jaw position, fit, and coupling are not trivial details, and unusual spikes should be interpreted with contextual information whenever possible [20,24,25]. Accordingly, the very highest PAA peaks in this dataset should be treated as hypothesis-generating signals for review (ideally with synchronised video), rather than as definitive indicators of true impact severity.

In applied terms, this supports three recommendations for future Muay Thai impact monitoring: (i) incorporate synchronised multi-angle video to interpret spikes and classify impact types; (ii) document mouthguard fit procedures and compliance; and (iii) explicitly report processing and labelling approaches so results are interpretable and comparable across studies, consistent with current iMG methodological guidance and video-verified field practice [17,20,21].

## 6. Conclusions

Instrumented mouthguards provide objective monitoring of head impact exposures in combat sports. In this case, sparring by a world-class athlete was predominantly low intensity with respect to linear acceleration; however, multiple sessions contained device-flagged severe angular-acceleration events. Regular impact monitoring may help coaches and fighters identify risky sparring exchanges, guide training adjustments, and preserve neurological health without compromising fight readiness.

## 7. Strengths and Limitations

A key strength of this case study is the use of a validated instrumented mouthguard (iMG) to capture in vivo head kinematics during real sparring sessions. Compared with helmet- or skin-mounted sensors, iMGs can reduce relative motion artefact and provide a practical approach for quantifying head impact exposure in unhelmeted combat sports, where training exposures are less well characterized than in many team collision sports. A further strength is the repeated monitoring across a complete fight camp, which enables description of how event frequency and magnitude distributions varied across sessions rather than relying on a single observation point.

Several limitations should be considered. First, this is a single-athlete case study, and findings are not intended to be generalized to other athletes, sparring partners, gyms, or training styles. Second, no concurrent clinical, symptom, or neurocognitive measures were collected, so the kinematic data cannot be linked to functional change or used to infer injury or concussion risk.

Third, no formal sparring-intensity scale was employed; intensity was described qualitatively by the athlete, reflecting the absence of standardised sparring-intensity criteria in Muay Thai and limiting the adjustment of impact exposure for session intensity.

Finally, and most importantly for interpretation, no independent video verification was performed. As a result, false positives or artefactual events could inflate both event counts and the apparent frequency of very high peak angular accelerations, particularly where jaw dynamics, mouthguard coupling, or transient sensor noise may influence peak estimates. This limitation is most relevant when interpreting clusters of high angular acceleration and any extreme peak values. For example, in Session 4, one event reached 162.2 g, which is substantially higher than other exposures in this dataset. Technical feedback from the manufacturer indicated that an open-mouth posture combined with clenching at the time of impact can exaggerate measured peaks. Therefore, while this event may reflect a true strike, the recorded magnitude may overestimate the underlying head kinematics. Similarly, “device-flagged severe-range classifications” based on peak thresholds should be interpreted cautiously in the absence of contextual confirmation, because device-specific processing, filtering, and windowing decisions can materially affect peak estimates and, in turn, severity banding.

For these reasons, the primary value of the present dataset is descriptive. The results are best interpreted at the level of event distributions within and between sessions (for example, medians, percentiles, and the proportion of events above pre-specified bands) rather than as definitive counts of confirmed high-severity impacts. Best practice for future work is to pair iMG outputs with multi-angle video verification to (1) confirm true impacts, (2) resolve suspected false positives, and (3) interpret unusually high or low peaks in relation to strike type, guard contact, and mouth posture.

A formal sensitivity analysis was not added because the investigators did not have access to raw time-series signals or alternative filtering and windowing parameters from the device, and therefore could not reprocess events under different signal-processing assumptions in a defensible way. Instead, we adopted a conservative interpretive approach by framing high-magnitude peaks and threshold-based categories as device-specific outputs and by avoiding causal language regarding injury risk or “device-flagged severe-range events” without video corroboration.

## 8. Practical Applications

The practical implication of this dataset is that sparring that feels “moderate” can still include rotationally substantial impacts. Given the mechanistic links between rotational kinematics and brain strain, training plans should prioritise reducing unnecessary rotational head motion while maintaining tactical and technical quality [8,9,10,12].

From a day-to-day coaching perspective, impact monitoring can be integrated into fight-camp programming in several practical ways:

**1.**  Session Structuring and Microcycle Planning
  - Schedule hard-contact sparring on clearly defined days rather than distributing high-intensity exchanges unpredictably across the week.
  - Follow higher angular-load sessions with technical or conditioning-focused sessions that
  - Minimize head contact.
  - Track weekly cumulative exposure to avoid clustering multiple high-load sessions within short time windows [3,4,5,7,23,28].
**2.**  Round-Level Adjustments
  - Cap the number of “open-intensity” rounds within a session when elevated angular peaks are observed.
  - Use rule modifications during camp (e.g., limited head strikes, controlled clinch intensity, situational drills) to preserve tactical development while reducing rotational loading.
  - Rotate sparring partners strategically to avoid repeated exposure to high-velocity or stylistically aggressive matchups in consecutive sessions.
**3.**  Technical Emphasis
  - Emphasise defensive positioning that limits head rotation, including controlled slips, compact guard recovery, and proactive head positioning in clinch.
  - Reinforce visual tracking and anticipation drills, as unexpected or “unseen” strikes may produce greater rotational excursions [8,9,23].
**4.**  Athlete Monitoring Integration
  - Use session summaries (e.g., total impacts, proportion above predefined bands, cumulative angular-load proxies) as an adjunct to traditional training-load metrics.
  - Identify “high brain-load” sessions and adjust subsequent volume or intensity accordingly, rather than reacting to single peak events.
  - Review unusually high peaks with contextual information (and ideally video) before modifying programming decisions [20,21].
**5.**  **Physical Preparation Considerations**
  - Incorporate structured neck-strength and neuromuscular control training as a potential mitigation strategy, while acknowledging that the magnitude of protective effect remains uncertain [8,9].
  - Integrate fatigue-management strategies, as technical breakdown late in sessions may increase uncontrolled rotational motion.

Overall, this case reinforces an important point for Muay Thai: angular kinematics add safety-relevant information that is not redundant with PLA, and rotationally dominated impacts can occur even when most PLA values appear low-to-moderate. When interpreted conservatively and in context, impact-monitoring data may assist coaches in refining sparring structure, sequencing high-intensity exposures, and managing cumulative head-impact load across a training camp.

## Figures and Tables

**Table 1:** Session-by-session summary.

Session	N Impacts	Mean PLA (g)	Max PLA (g)	PLA ≥ 66 g	PLA > 106 g	Mean PAA (rad/s^2^)	Max PAA (rad/s^2^)	PAA ≥ 4600 (rad/s^2^)	PAA ≥ 7900 (rad/s^2^)
1	10	11.1	28.4	0	0	1028.8	3776.1	0	0
2	100	7.6	19.0	0	0	529.6	6839.1	1	0
3	86	9.2	67.3	1	0	1097.9	10,863.1	1	1
4	123	19.5	162.2	3	3	2784.0	19,862.5	9	9
5	60	10.2	58.8	0	0	971.5	6158.4	1	0
6	129	15.6	101.0	1	0	1893.4	26,850.2	2	2
7	82	16.8	118.0	1	1	2180.2	18,677.5	2	2

**Abbreviations:** PLA, peak linear acceleration (g); PAA, peak angular acceleration (rad/s^2^); N, number of detected impacts (events). PLA severity bands were defined as mild (<66 g), moderate (66–106 g), and device-flagged severe range (>106 g). PAA severity bands were defined as mild (<4600 rad/s^2^), moderate (4600–7900 rad/s^2^), and device-flagged severe range (>7900 rad/s^2^).

## Data Availability

The data presented in this study are available from the corresponding author upon reasonable request. The data are not publicly available due to privacy and confidentiality considerations, as they contain participant-level health information that could potentially enable identification. Data sharing will be considered in accordance with participant consent and applicable privacy regulations.
